# Emotional self-awareness in autism: A meta-analysis of group differences and developmental effects

**DOI:** 10.1177/1362361320964306

**Published:** 2020-11-05

**Authors:** Charlotte F Huggins, Gemma Donnan, Isobel M Cameron, Justin HG Williams

**Affiliations:** University of Aberdeen, UK

**Keywords:** alexithymia, autism, development, emotional awareness, mental health

## Abstract

Emotional self-awareness is increasingly suggested to be an area of difficulty in autism that may predict socioemotional outcomes for this population. However, whether emotional self-awareness is consistently diminished in autism across age and methodology remains unclear. We systematically reviewed 47 papers measuring emotional self-awareness in autistic (*n* = 1387) and non-autistic (*n* = 1433) participants. Most studies relied on self-report. Of studies testing for group differences, the majority (32/41) found significantly poorer emotional self-awareness in autism. Meta-analyses of self-report measures found that emotional self-awareness was significantly poorer in autism (*d* = 1.16). However, when examining age groups individually, autistic children of age 12 years and under were not significantly different from their peers (*d* = 0.03). Instead, difficulties emerged during adolescence (*d* = 0.63) and increased with age (*d* = 1.16 – 1.58). The pattern of emotional self-awareness difficulties being more common in autism, and worsening with age is similar to the development of mental health difficulties in autism. However, findings rely on self-perception and so may reflect poor self-beliefs of socioemotional competence. We propose that negative self-beliefs in autistic populations may account for findings of low emotional self-awareness.

**Lay abstract:**

Autistic people are thought to have difficulties with identifying and understanding their own emotions. This is referred to as *emotional self-awareness.* It is important to study emotional self-awareness as people who are more able to understand their own emotions, whether they are autistic or not, are more able to respond to them appropriately, as well as to identify them in other people. It has not yet been confirmed whether autistic people have difficulties with emotional self-awareness, or if any reported difficulties are actually due to the way in which emotional self-awareness is measured in autistic people. If these difficulties do exist, it is also not known when these difficulties emerge. In this research, we reviewed 47 existing studies that measured emotional self-awareness in autistic and non-autistic adults and children. We also compared studies that measured emotional self-awareness in different ways. We found that autistic adults did seem to have poorer emotional self-awareness compared to their neurotypical peers. However, this was not the case with autistic children of age 12 years and below. Instead, differences in emotional self-awareness only seemed to emerge during adolescence. Moreover, these difficulties seemed to increase with age. These results suggest that difficulties with emotional self-awareness may not be inherent in autism. Instead, they may emerge alongside the greater social and mental health difficulties that are experienced by many autistic people during adolescence. We therefore suggest that it is important to find out more about, and subsequently support, the emotional self-awareness difficulties that autistic adolescents may encounter.

An important goal of clinical practice in autism is improving an individual’s social functioning. One key target for this process may be the ability to describe and identify one’s own emotions. While many terms have been used to describe this ability (Kashdan et al., 2015), for this review, we use the term ‘emotional self-awareness’. Recent review work suggests that emotional self-awareness is diminished in autism (Kinnaird et al., 2019) and that these difficulties predict poorer emotional contagion and recognition in this population (Bird & Cook, 2013).

This is the second part of a two-part review. In the first review (Huggins et al., 2020), we qualitatively reviewed how emotional self-awareness was conceptualised, defined and measured in autism research, finding that emotional self-awareness is usually assessed with self-report, and tends to be inconsistently defined. In this review, we examine group differences in emotional self-awareness across the different measurement tools between autistic and non-autistic groups with meta-analysis. We also examine developmental patterns of emotional self-awareness.

A long-standing theory is that the social difficulties of autism are rooted in delayed development of ‘Theory of Mind’ (ToM)–the ability to represent the mental states of oneself and others (Baron-Cohen, 1995; Perner et al., 1989). Although studies consistently find that autistic children struggle to report their own mental states (Williams, 2010), most research has focused on understanding the minds of others. However, the ability to represent one’s own mind may be fundamental to representing the minds of others (Decety & Meyer, 2008), making emotional self-awareness an increasing area of interest in autism research.

Much research on emotional self-awareness in autism has focused on alexithymia (Poquerusse et al., 2018). Alexithymia was originally used to describe the behaviours of psychosomatic patients who struggled to communicate their own emotions, tending to focus on external stimuli over subjective internal experiences (Sifneos, 1973). In contemporary research, alexithymia is frequently conceptualised as a trait reflecting difficulties in identifying and describing one’s own emotional experiences, usually assessed with self-report (Moriguchi & Komaki, 2013).

Recent meta-analytic work demonstrated significantly elevated alexithymia in autism (Kinnaird et al., 2019). A significant limitation of this review is that it exclusively surveyed studies using the 20-item Toronto Alexithymia Scale (TAS-20; Bagby et al., 1994; Taylor et al., 1985), a self-report questionnaire. Although it is one of the most commonly used, well-validated measures of alexithymia, the TAS-20 has some limitations.

TAS-20 scores are strongly associated with general negative affect (Lumley, 2000) and depression (Honkalampi et al., 2001). Longitudinal work demonstrates that TAS-20 scores fluctuate with general mental health (Marchesi et al., 2008, 2014). It has thus been suggested that the TAS-20 measures general psychological distress, rather than a stable trait (Leising et al., 2009). Moreover, there is a high prevalence of mental health conditions in autism (Simonoff et al., 2008; Lai et al., 2019). As such, greater psychological distress could account for the elevated TAS-20 scores in this population. Alternatively, poor emotional self-awareness, as measured by TAS-20 scores, may result in greater difficulties regulating negative emotion and thus elevated psychological distress. Regardless, psychological distress remains a potential confounding factor in this relationship.

Furthermore, though self-report can provide a valuable source of information on subjective experiences, it is reliant upon self-perception. Even in the general population, self-report and performance-based measures of emotional abilities correlate weakly (Keefer, 2015; Lumley et al., 2005). This is unsurprising given that self-report relies on comparing one’s own experiences and abilities to those of others, which is largely impossible in emotional experience. People with poor emotional self-awareness are also likely to have poor awareness of their abilities (Marchesi et al., 2014). Difficulties with judging one’s own abilities may be particularly problematic in autism, due to the greater meta-cognitive difficulties seen in this population (Garfinkel et al., 2016; Grainger et al., 2014; Palser et al., 2018).

The focus on ‘alexithymia’ also overlooks similar constructs which have arisen to measure emotional self-awareness (Kashdan et al., 2015). Alexithymia is largely conceptualised as a deficit based on behaviours in clinical populations (Sifneos, 1973). Other definitions conceptualise emotional self-awareness as an ability, such as in emotional intelligence (Salovey & Mayer, 1990), and do not contain externally-oriented thinking. For instance, emotion differentiation focuses specifically on how well an individual differentiates between similar emotional experiences (Barrett et al., 2001). The overlap between these similar concepts remains poorly understood. Throughout this article, we use ‘emotional self-awareness’ as an umbrella term. When referring to outcomes from a measure, we will use the term specified by that measure (e.g. referring to TAS-20 scores as representing ‘alexithymia’). Examining whether emotional self-awareness is diminished in autism across methodologies and approaches would help us to determine whether current findings arise from use of the TAS-20.

Another question raised by Kinnaird et al. (2019) is whether emotional self-awareness is affected by development. They found that increasing age was associated with greater alexithymia, across both autistic and non-autistic groups and suggested this may result from greater rates of depression and anxiety in adulthood. In the general population, increasing age is associated with poorer emotional self-awareness (Mattila et al., 2006), and such a decline may be particularly pertinent in autism. However, Kinnaird et al. examined how alexithymia levels change with age across groups, but not changes in the difference *between* groups.

Addressing when emotional self-awareness difficulties arise may also help pinpoint the aetiology of such problems. If difficulties with emotional self-awareness are widely present from an early age, emotional self-awareness difficulties may be linked to fundamental issues in autism, such as delayed ToM (Happé, 1995). However, if emotional self-awareness difficulties emerge at a later point, this would suggest that they stem from other causes, such as co-occurring mental health problems.

We aimed to (a) assess if there are significant impairments in emotional self-awareness in autism when measured with different measurement tools, including those outside of targeting emotional self-awareness concepts outside of alexithymia, and (b) assess how emotional self-awareness changes during development in autistic and typical development groups.

## Methods

The current review followed PRISMA guidelines for systematic reviews. The protocol for the current review is registered on the PROSPERO database, identification number CRD42017082052 (available online at https://www.crd.york.ac.uk/PROSPERO/display_record.php?RecordID=82052), and is based on literature published to May 2018. Qualitative comparisons of measurement tools and term definitions are available in a separate paper (Huggins et al., 2020). Please note that this review is based on the same literature as in this first paper (Huggins et al., 2020), and as such, the search strategy and eligibility criteria remain the same.

### Eligibility criteria

Inclusion criteria were as follows: (a) participants had a diagnosis of autism spectrum disorder (ASD), including Autism, Asperger’s, Pervasive Developmental Disorder–Not Otherwise Specified (PDD-NOS), and autism spectrum condition (ASC); (b) the study included a healthy, non-autistic control group; (c) the study included at least one measure *explicitly* assessing the participant’s awareness of their own emotional states, which is clearly distinct from measures assessing ability to identify or describe others’ emotional states.

Items were also excluded if (a) study was not published in English or (b) study was not an empirical paper in a peer-reviewed journal.

## Search strategy

Searches were conducted on the databases Scopus, Web of Science, ScienceDirect, PsycARTICLES, Embase, Medline and PsychINFO, across all published reports until May 2018.

Databases were searched for any articles with any combination of the following keywords in the Title, Abstract and Keywords: (1) ‘autism’ or ‘ASD’ or ‘ASC’ or ‘Autism Spectrum Disorder’ or ‘Autism Spectrum Condition’ or ‘autistic’ or ‘Asperger’ or ‘PDD-NOS’ or ‘Pervasive Developmental Disorder Not Otherwise Specified’, and (2) ‘alexithymia’ or ‘emotional awareness’ or ‘emotional differentiation’ or ‘emotion differentiation’ or ‘emotional granularity’ or ‘emotional intelligence’ or ‘emotional competence’ or ‘emotion labelling’ or ‘emotional labelling’.

**Figure 1.**
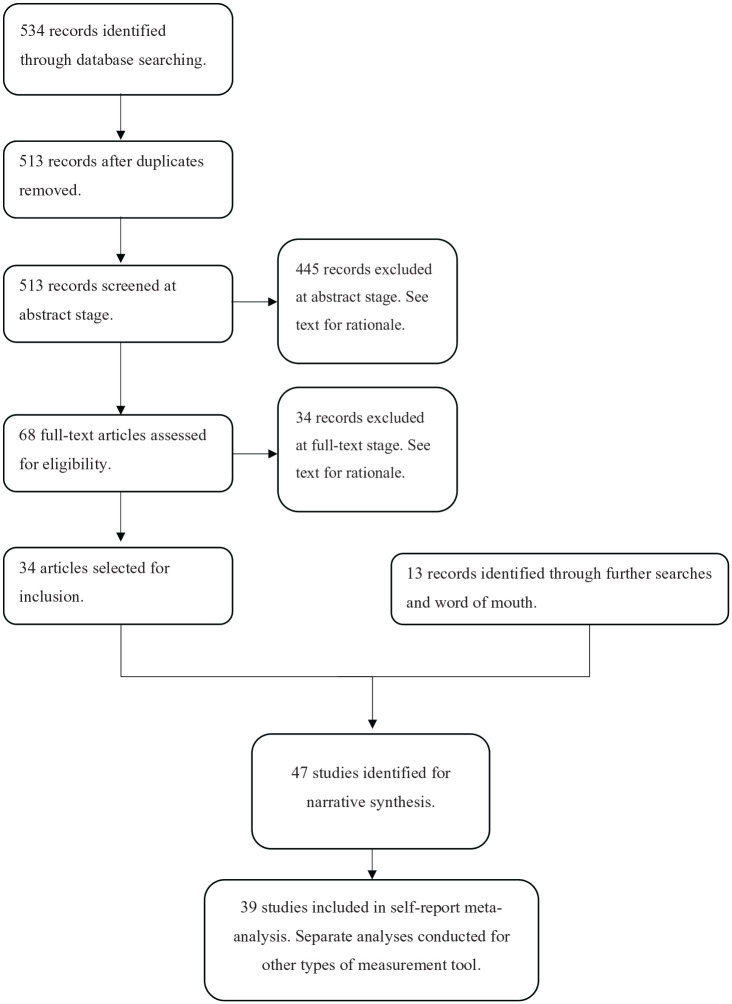
PRISMA flowchart of search process.

Interoception, while undoubtedly relevant to emotional self-awareness on the theoretical level (Craig, 2003), is not a direct measure of subjective awareness of one’s own emotional states and was not included in search strategy. PRISMA flowchart of the search process can be seen in Figure 1.

### Study selection and exclusions

#### Stage 1

Following exclusion of duplicate articles, 513 items were identified for abstract and title screenings. Screening was conducted by C.F.H. and G.D. separately. The results of these separate screenings were collated, and areas of disagreement were resolved through discussion. Of excluded papers, 21 papers were in a language other than English, 180 were not empirical papers, 174 did not include an autistic sample, 27 did not include non-autistic controls and 43 did not include an explicit measure of emotional self-awareness. The final list of papers for full-text screenings was 68.

After full-text screenings, 16 studies did not include any direct measure of emotional self-awareness, 5 were student dissertations, 2 did not have appropriate controls, 1 was a conference abstract, 1 was a study protocol and 1 was not in English. A further eight papers were excluded from as participants were matched by alexithymia. A final 34 papers were agreed upon by both researchers. A standardised data form was used to extract data.

#### Stage 2

Initial analyses confirmed the TAS-20 as the most frequently used measurement tool. Further searches were conducted to identify any studies missing from the current data set using the TAS-20 that did not mention it in the title, abstract or key words.

Databases were searched for any articles with a combination of terms indicative of autism (see (1) above) ‘Toronto Alexithymia Scale’ OR TAS-20. Following exclusion of duplicate items, 261 items were identified. After screening and full-text review, 11 additional papers were identified for inclusion.

Due to the high amount of papers identified this way, full-text searches were conducted for all identified measurement tools. All search terms combined with previously used autism terms. Searches were completed in May 2018. Across these additional searches, only one additional paper was identified for inclusion and one additional paper was further identified by word of mouth. The final data set consisted of 47 papers, which can be seen in Supplementary Information B, Table 1.

Means and standard deviations of emotional self-awareness scores and subscales were extracted. The number of participants in each sample with TAS-20 total scores of 61 or over were also extracted. To control for the effect of drop-outs and avoid replication in the data set, only scores from the final time point were extracted in longitudinal studies. Authors were contacted for missing data. If no response was available after contacting the named contact author and the first or second author twice, data were coded as missing.

### Quality assessment

The quality of each paper was assessed quantitatively with a custom assessment form (see Supplementary Information B, Figure 1), scored from 0 (poor quality) to 19 (excellent quality). Quality assessment scores ranged from 2 to 15, with a mean score of 7.83 (SD = 2.61). Scores for each paper can be seen in Supplementary Information B, Table 1. Further details of quality assessment and risk of bias can be seen in Supplementary Information A.

## Results

### Participant information

Across all 47 studies, a total of 2820 participants (23.89 % female) were included in analyses. Of these participants, 1387 had a diagnosed autism spectrum disorder (21.12% female), compared to 1433 with no such diagnosis (25.89% female). In 14 of the 47 studies, diagnoses were confirmed with both Autism Diagnostic Interview (ADI) and Autism Diagnostic Observation Schedule (ADOS), and a further 8 used either the ADI or ADOS. No studies were based on autistic traits in typical populations. Participant ages ranged from 3 to 80 years. Most studies examined adults (33; mean age = 30.74 (5.56)), 7 examined adolescents (mean age = 15.17 (0.83)) and 7 examined children (mean age = 30.74 (5.56)). Specific data on race and socioeconomic status were not available in all studies. Outline of recruitment methods can be seen in Supplementary Information A.

### Group differences

Only 6 papers in this data set of 47 studies did not explicitly test for group differences in emotional self-awareness. Of the remaining 41 papers, 32 found significantly poorer emotional self-awareness in autism at α = 0.05. Of the remaining nine papers, two papers only examined TAS-20 subscales and four papers found mixed results with multiple tools (Arellano et al., 2018; Berthoz & Hill, 2005; Erbas et al., 2013; Silani et al., 2008). In two of these studies, the TAS-20 found significantly higher scores among autistic compared to typical populations, while the Bermond–Vorst Alexithymia Questionnaire (BVAQ) found no difference (Berthoz & Hill, 2005; Silani et al., 2008). Similarly, Arellano and colleagues (2018) found TAS-26 scores were significantly higher in autistic populations, but no differences emerged in BVAQ scores. Findings from Erbas and colleagues (2013) are explored in the ‘Behavioural measures’ section. Only five papers found no statistically significant differences in emotional self-awareness between groups when testing for this.

Most (42/47) papers exclusively used self-report measures. Others used parent-report measures (2), behavioural measures (2), and a combination of self-report and parent-report measures (1). For details, see Supplementary Information B, Table 2.

Of the 43 studies that used self-report measures, 29 used the TAS-20. A 2 (TAS-20 vs non-TAS-20) × 3 (Significant difference vs No significant difference vs Not test) Chi-square tests found studies using the TAS-20 were not significantly more likely to find significant differences compared to other methods, χ^2^ = 4.7483, *p* = 0.093.

### Self-report measures

Overall group differences in self-report outcomes were determined using a random-effects meta-analysis. Mean values and standard deviations were available for 39 data sets, with 2480 participants (1206 autistic; 1274 typical controls). Papers were grouped by measure used. To match TAS-20 scores, scores were coded so that higher scores reflected greater emotional self-awareness difficulties. Notably, six studies used more than one self-report measure, leading to some samples of participants being included twice (Allen et al., 2013; Arellano et al., 2018; Berthoz et al., 2013; Berthoz & Hill, 2005; Silani et al., 2008).

Meta-analyses demonstrated autistic participants has significantly greater emotional self-awareness difficulties across self-report outcomes, *Z* = 10.62, standard mean difference (SMD) = 1.16, 95% CI = [0.94, 1.37], *p* < 0.001. There was a very high, statistically significant amount of heterogeneity across the data set, χ^2^ = 243.69, *I*^2^ = 82%, *p* < 0.001. Moreover, there were significant differences between tools, χ^2^ = 30.62, *I*^2^ = 83.7%, *p* < 0.001, indicating that effect sizes differed depending on the measurement tool used. Therefore, analyses for each tool were examined separately. For sake of comparison across tools, SMDs were used in each case.

#### TAS-20

**Figure 2.**
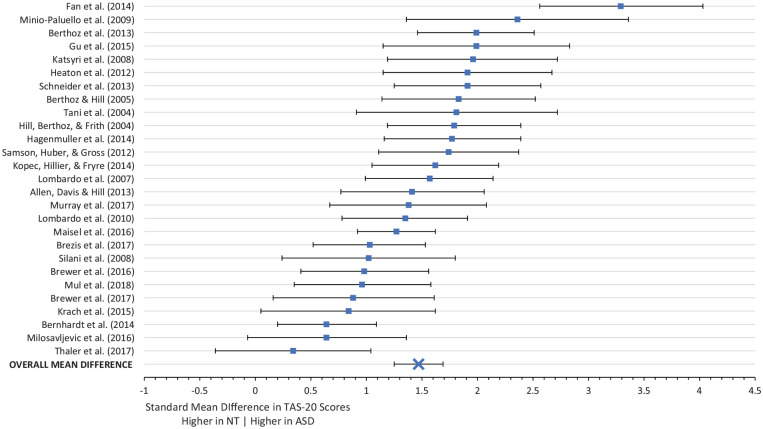
Forest plot of standard mean differences in TAS-20 total scores between ASD and NT participants.

Of the 29 papers assessing group differences in TAS-20 scores, 27 examined adults and 2 examined adolescents. Two papers did not provide required data and were thus excluded from analyses. A significant difference emerged between autistic (*n* = 702, M = 57.61, SD = 11.80) and non-autistic (*n* = 731, M = 42.27, SD = 9.50) participants in total TAS-20 scores, *Z* = 12.96, SMD = 1.47, 95% CI = [1.24, 1.69], *p* < 0.001. There was a moderate amount of heterogeneity, χ^2^ = 85.39, *I*^2^ = 70%, *p* < 0.001. SMD between TAS-20 scores by group ranged from 0.34 to 3.29; see Figure 2 for forest plot.

The TAS-20 has three subscales: Difficulty Identifying Feelings (DIF), Difficulties Describing Feelings (DDF) and Externally Oriented Thinking (EOT). The DIF subscale assesses ability to identify one’s own emotions and distinguish these from bodily sensations. The DDF subscale assesses ability to describe and communicate feelings to others. Finally, EOT assesses tendency to focus on external stimuli, rather than internal experiences.

DIF subscale (14 studies) scores were significantly different between autistic (*n* = 446, M = 19.41, SD = 5.77) and non-autistic (*n* = 441, M = 12.96, SD = 4.59), *Z* = 9.31, SMD = 1.16, 95% CI = [0.92, 1.40], *p* < 0.001. There was a moderate, significant amount of heterogeneity, χ^2^ = 33.95, *I*^2^ = 62%, *p* < 0.001.

A significant difference also emerged between autistic (*n* = 446, M = 16.53, SD = 4.52) and non-autistic (*n* = 441, M = 11.09, SD = 3.96) participants in DDF subscale (14 studies) scores, *Z* = 10.87, SMD = 1.20, 95% CI = [0.098, 1.41], *p* < 0.001. There was a moderate amount of heterogeneity, χ^2^ = 26.33, *I*^2^ = 51%, *p* < 0.001.

A significant difference emerged in EOT subscale (14 studies) scores between autistic (*n* = 446, M = 20.69, SD = 4.50) and non-autistic (*n* = 441, M = 16.88, SD = 4.18) participants, *Z* = 7.16, SMD = 0.88, 95% CI = [0.64, 1.12], *p* < 0.001. There was a moderate amount of heterogeneity, χ^2^ = 35.45, *I*^2^ = 63%, *p* < 0.001.

**Figure 3.**
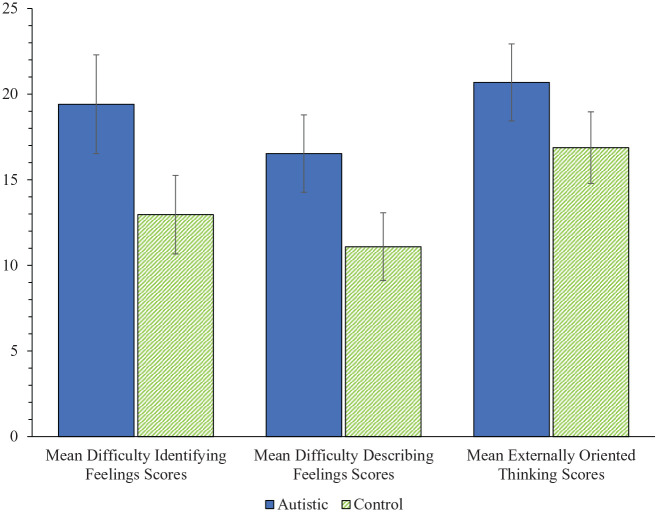
Average TAS-20 subscale scores across studies in autistic and non-autistic samples.

Participants with autism scored higher on average across all three subscales, with the magnitude of difference greatest for the DIF subscale and smallest for the EOT subscale (see Figure 3).

##### Alexithymia prevalence

Overall prevalence of ‘severe alexithymia’ was compared in autistic and non-autistic participants with meta-analysis of odds ratios. Alexithymia was classified as ‘severe’ if TAS-20 scores were 61 or above, in line with previous research (Parker et al., 2003). Prevalence data were available for 18 out of 29 TAS-20 studies. Autistic participants were significantly more likely to have severe alexithymia – 204 (41.0%) of the 497 autistic participants had TAS-20 scores of 61 or above, compared to only 25 (4.9%) of the 508 non-autistic participants, Odds ratio = 10.58, 95% CI = [5.75, 19.45], *p* < 0.0001, with no significant heterogeneity, χ^2^ = 50.69, *I*^2^ = 36%, *p* = 0.07

#### 26-item Toronto Alexithymia Scale

Only two studies in the current data set used the TAS-26. A random-effects meta-analysis found TAS-26 scores were significantly higher in autistic participants (*n* = 29, M = 48.70, SD = 7.33), compared to non-autistic participants (*n* = 38, M = 35.96, SD = 5.64), *Z* = 2.00, SMD = 1.82, 95% CI = [0.04, 3.60], *p* = 0.05. Heterogeneity was significant and high, χ^2^ = 8.18, *I*^2^ = 88%, *p* = 0.004.

#### BVAQ

There are three forms of the BVAQ: form A, B, and AB (Vorst & Bermond, 2001). Forms A and B are 20 items long. Items presented on the A and B form differ, but both have the same number of items for each subscale. Form AB is a compilation of both forms, composing of 40 items. Of the nine studies included in the current analysis, four used the BVAQ-AB, and five used the BVAQ-B. No studies used the BVAQ-A. One study (Duijkers et al., 2014) was excluded for not including BVAQ average scores for the typically developing comparison group.

**Figure 4.**
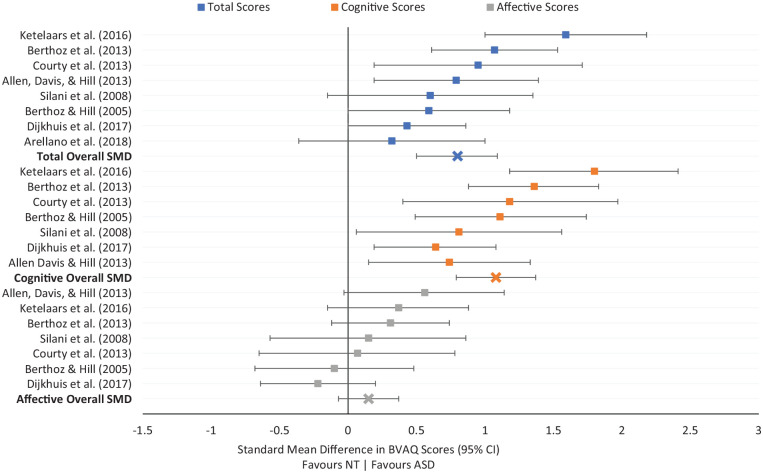
Forest plot of standard mean differences between autistic and non-autistic participants in BVAQ total and component scores.

A random-effects meta-analysis was conducted. See Figure 4 for a forest plot of comparisons.

Total BVAQ scores were significantly higher in autistic (*n* = 235) compared to non-autistic (*n* = 208) participants, *Z* = 5.33, SMD = 0.80, 95% CI = [0.50, 1.09], *p* < 0.001. No significant differences between studies using the BVAQ-AB and the BVAQ-B were found, χ^2^ = 0.02, *p* = 0.88, indicating there was no interaction between-group differences and which BVAQ version is used. A moderate amount of heterogeneity was found, χ^2^ = 13.94, *I*^2^ = 50%, *p* = 0.05.

Cognitive BVAQ scores were significantly higher in autistic (*n* = 221) compared to non-autistic (*n* = 187) participants, *Z* = 6.61, SMD = 1.08, 95% CI = [0.76, 1.40], *p* < 0.001. No differences between studies using the BVAQ-AB and the BVAQ-B were found, χ^2^ = 0.03, *p* = 0.85. A significant amount of moderate heterogeneity was found, χ^2^ = 12.39, *I*^2^ = 52%, *p* = 0.05.

No significant effect of group was found in affective BVAQ scores (autistic, *n* = 221, non-autistic, *n* = 187), *Z* = 1.38, SMD = 0.15, 95% CI = [−0.06, 0.377], *p* = 0.17. No differences between studies using the BVAQ-AB and BVAQ-B were found, χ^2^ = 0.27, *p* = 0.60, and no statistical heterogeneity was detected, χ^2^ = 6.75, *I*^2^ = 11%, *p* = 0.34.

#### Bar-On Emotional Quotient Inventory–Intrapersonal Scale

Of the four studies that use the Bar-ON EQ-I, intrapersonal subscale scores were available for three studies. Random-effects meta-analyses found no significant differences between groups (autistic, *n* = 72, M = 96.51, SD = 16.09; non-autistic, *n* = 72, M = 96.23, SD = 12.84), *Z* = 0.09, SMD = 0.03, 95% CI = [−0.83, 0.78], *p* = 0.93. Statistical heterogeneity was significant and high, χ^2^ = 10.84, *I*^2^ = 82%, *p* = 0.004.

#### Emotional Awareness Questionnaire–Differentiation Subscale

The Emotional Awareness Questionnaire–Differentiation Subscale (EAQ-diff) was used in two studies. A random-effects meta-analysis found no significant difference in EAQ-diff between groups (autistic, *n* = 122, M = 2.15, SD = 16.09; non-autistic, *n* = 174, M = 2.35, SD = 12.84), *Z* = 1.21, SMD = −0.18, 95% CI = [−0.95, 0.23], *p* = 0.23. Heterogeneity was high and significant, χ^2^ = 5.90, *p* < 0.001, *I*^2^ = 83%.

#### Children’s Alexithymia Questionnaire

Two studies use the Children’s Alexithymia Questionnaire (CAQ). A random-effects meta-analysis found CAQ did not significantly differ between groups (autistic, *n* = 45, M = 18.89, SD = 16.09; non-autistic, *n* = 52, M = 15.55, SD = 12.84), *Z* = 1.83, SMD = 0.60, 95% CI = [−0.004, 1.24], *p* = 0.07. Heterogeneity was not statistically significant, χ^2^ = 2.38, *p* = 0.12, *I*^2^ = 58%.

### Other-report measures

In the three studies which used other-report measures (Costa et al., 2017; Griffin et al., 2016; Trevisan et al., 2016), two measurement tools were identified: the Children’s Alexithymia Measure (CAM-PR; Way et al., 2010) and the Alexithymia Questionnaire for Children (ACQ-P; Costa et al., 2017). Both were specifically intended for use with children, to be filled in by the parents or carers.

The CAM-PR (Way et al., 2010) is a 14-item observer-report measure of alexithymia, intended for children between ages 5 and 17 years. Initial validity work found the CAM-PR has high internal consistency and good concurrent validity with other measures of alexithymia, producing a unidimensional measure of alexithymia in children. The CAM-PR was developed through focus group work, expert panel feedback, and psychometric analysis of questionnaire data.

The ACQ-P (Costa et al., 2017) is a 20-item observer-report measure of alexithymia. The ACQ-P is an adaptation of the self-report CAQ (Rieffe et al., 2006), a version of the TAS-20, with the questions reformulated to be parent-report items. The ACQ-P shows good internal consistency, and a three-factor structure in line with the TAS-20 (Costa et al., 2017).

Two studies in the current data set utilised the CAM, and one used the ACQ-P. All three studies were compared with random-effects meta-analysis. Autistic participants (*n* = 79) had significantly more emotional self-awareness difficulties compared to non-autistic participants (*n* = 90), *Z* = 6.30, SMD = 1.26, 95% CI = [0.87, 1.65], *p* < 0.001. No significant heterogeneity was detected, χ^2^ = 0.35, *p* = 0.56, *I*^2^ = 0%.

### Behavioural measures

Three behavioural measures were identified in two papers: the Multiple Emotions Task (MET; Rieffe et al., 2007), Photo Emotion Differentiation Task (PED-task; Erbas et al., 2013), and the Emotion Sorting Task (ES task; Erbas et al., 2013).

In the MET, participants listen to a story intended to elicit emotion, paired with a simple illustration. Participants are invited to select the emotion that they would feel in each situation. In the PED-task, participants view emotional images paired with various negative emotional terms, rating how strongly each image evoked each discrete emotional state. Differentiation was calculated by examining correlations between emotion ratings. In the ES task, participants were asked to sort 20 negative emotional words into groups according to how the words ‘belong together’. Differentiation was quantified through number of groups that the participants divided words into. These measurement tools were judged as too different to conduct meaningful meta-analytic comparisons.

Rieffe and colleagues (2007) found that children with autism identified significantly fewer emotional perspectives per story compared to their typically developing counterparts on the MET, *F*(1, 42) = 4.64, *p* = 0.03. Erbas and colleagues (2013) found that participants with autism showed significantly less emotional differentiation than typically developing participants in the PED-task, *U* = 107.00, *p* = 0.04, but this only approached statistical significance in the ES task, *U* = 114.00, *p* = 0.06.

### Comparisons by age group

**Figure 5.**
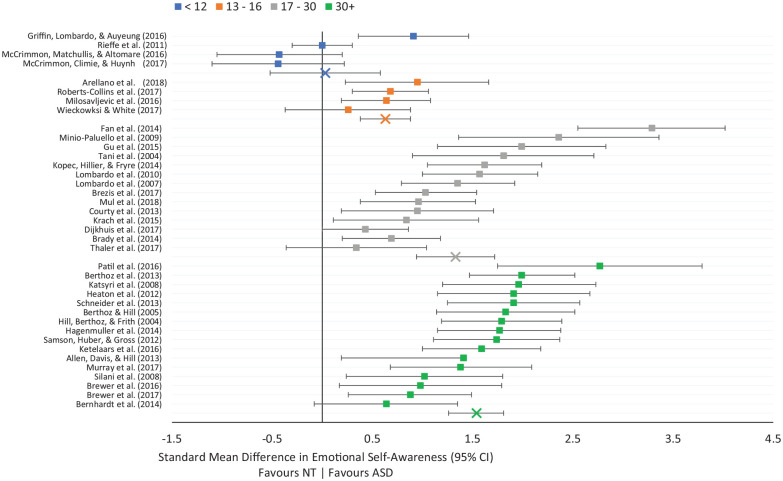
Forest plot of standard mean differences in emotional self-awareness across age ranges.

To assess whether emotional self-awareness may change over time, group differences were compared at four different age ranges: 12 years and under (8 studies), 13–16 years (6 studies), 17–30 years (16 studies), and 31 years or over (18 studies). Studies were categorised based on the average age of the autistic sample. Only self-report measures were used, to control for the effects of differing methodologies, and studies where age data were not available were excluded. Consequently, 38 studies were included in the final analyses (12 years and under = 4 studies; 13–16 years = 4 studies; 17–30 years = 14 studies; 31+ years = 16 studies). See Figure 5 for forest plot.

In children 12 years old and younger, no differences between autistic (*n* = 129) and non-autistic participants (*n* = 188) were found, *Z* = 0.10, SMD = 0.03, 95% CI = [−0.52, 0.58], *p* = 0.92. Heterogeneity was both high and significant, χ^2^ = 13.80, *I*^2^ = 78%, *p* = 0.006.

In adolescents of age 13–16 years, a significant, moderate effect size of group was found, *Z* = 5.01, SMD = 0.63, 95% CI = [0.38, 0.88], *p* < 0.001. Autistic participants (*n* = 146) showed significantly less emotional self-awareness compared to non-autistic participants (*n* = 129). No significant statistical heterogeneity was observed, χ^2^ = 2.21, *I*^2^ = 0%, *p* = 0.53.

A large, significant effect of group emerged in the studies assessing self-report emotional self-awareness in autistic (*n* = 390) and non-autistic (*n* = 353) participants of age 17–30 years, *Z* = 6.67, SMD = 1.33, 95% CI = [0.94, 1.72], *p* < 0.001. Heterogeneity was large and significant, χ^2^ = 69.96, *I*^2^ = 67%, *p* < 0.0001.

Finally, a large and significant difference emerged between autistic (*n* = 356) and non-autistic (*n* = 393) in the age 31 and above group, *Z* = 13.31, SMD = 1.58, 95% CI = [1.35, 1.82], *p* < 0.001. Statistical heterogeneity was significant but moderate, χ^2^ = 28.20, *I*^2^ = 47%, *p* = 0.02. Differences between age groups were significant, χ^2^ = 46.22, *I*^2^ = 93.5%, *p* < 0.001. These findings suggest that age is associated with increasing disparity in emotional self-awareness between autistic and non-autistic groups. Correlation analyses align with this, finding that SMD scores were significantly correlated with mean age of participants, *r*(35) = 0.672, *p* < 0.001.

**Figure 6.**
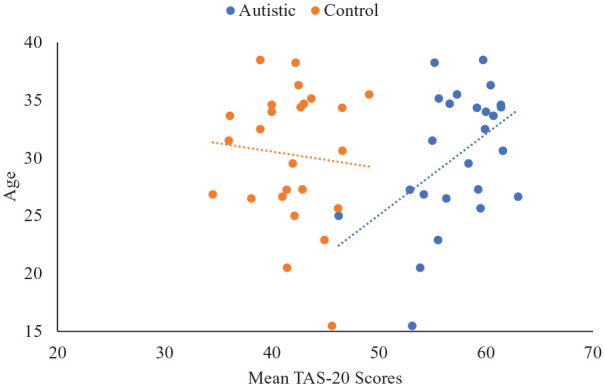
Scatterplot showing average age of group against mean emotional self-awareness difficulties.

We next assessed whether changes in emotional self-awareness with age were associated with changes in autistic or typical groups. As measurement tools were not comparable, this analysis was done with TAS-20 scores only. Correlation analyses compared mean TAS-20 scores for autistic and non-autistics groups to overall mean age score in the study. Among autistic participants, greater mean age was associated with greater TAS-20 scores, *r*(23) = 0.456, *p* = 0.022. No significant association between age and TAS-20 scores emerged for the typical controls, *r*(23) = −0.089, *p* = 0.672 (see Figure 6). These findings suggest that emotional self-awareness worsened with age among the autistic but not typical populations.

## Discussion

Emotional self-awareness is increasingly recognised as an important predictor of socioemotional outcomes, particularly in autism. Despite this, there is still little understanding of how and when emotional self-awareness difficulties arise, and how methodological biases influence these outcomes. We first replicated Kinnaird et al.’s (2019) findings that emotional self-awareness is diminished in autism. However, the strength of this effect varied by measurement tool, with the strongest effect sizes found in studies using the TAS-20. Furthermore, as we illustrated in the first part of the review (Huggins et al., 2020), few studies used observer-report or behavioural methods, further details can be seen in first part of review.

We identified divergent developmental patterns of emotional self-awareness in autistic and non-autistic groups. While no differences emerged in pre-adolescent children, differences emerged during adolescence and increased with age. Moreover, correlational analyses suggest that this relates to declining abilities in the autistic group, rather than increasing ability in the non-autistic group. This developmental pattern bears a resemblance to other developmental patterns in autism, such as facial emotion recognition (Lozier et al., 2014) and eye-gaze (Black et al., 2017).

Interoceptive accuracy likewise decreases with age in autistic populations (Mash et al., 2017), albeit only in participants with IQs lower than 115. Interoceptive abilities are fundamental to forming a conscious awareness of one’s own emotions (Craig, 2003; Seth, 2013). Thus, declining emotional self-awareness in autism may stem from increasing interoceptive difficulties. However, this does not explain why such difficulties should arise with age. One possibility is that autistic people may develop greater awareness of their difficulties with age and thus become more likely to report poorer emotional self-awareness.

Another possible explanation is the prevalence of comorbid mental health difficulties in autism (Lai et al., 2019; Simonoff et al., 2008). Poorer emotional self-awareness is associated with depression (Demiralp et al., 2012) and anxiety (Kashdan & Farmer, 2014). The TAS-20 tends to be associated with general negative affect (Lumley, 2000), and has been suggested to measure psychological distress rather than a stable trait (Leising et al., 2009), suggesting that emotional self-awareness may fluctuate with mental health.

During adolescence and adulthood, autistic symptoms tend to abate, while depression and anxiety symptoms often become more severe (Anderson et al., 2011). The gap in social skills between autistic children and their peers also widens during adolescence (Church et al., 2000), increasing the risk of social isolation or victimisation. Such difficulties likely continue into adulthood, with autistic adults less likely to be employed or in higher education (Wehman et al., 2014). This social isolation deprives autistic people of social and emotional learning opportunities compared to their peers, contributing to impaired development of social cognitive abilities, including emotional self-awareness. Furthermore, these increased mental health issues and greater social isolation are likely to result in diminished self-beliefs in socioemotional competence.

Few studies in our review controlled for comorbid mental health issues. As such, it remains difficult to examine the extent to which emotional self-awareness difficulties relates to autism itself, or mental health problems in this population. Future research may benefit from controlling for differences in depression and anxiety symptoms between groups.

In addition, few studies controlled for cognitive ability and verbal skills. Previous research suggests that greater alexithymia is associated with lower verbal IQ (Montebarocci et al., 2011), and greater verbal ability predicts greater emotion differentiation (Israelashvili et al., 2019). As such, emotional self-awareness may also depend on general cognitive ability, as well as emotional vocabulary. However, the majority of these studies focused on populations with average-to-high cognitive ability, limiting variability. Nevertheless, future research should examine the extent to which vocabulary or cognitive ability predicts emotional self-awareness in autism.

While self-report data indicates that pre-adolescent autistic children did not differ from their peers in emotional self-awareness, parent-report outcomes found that autism was associated with poorer emotional self-awareness (Costa et al., 2017; Griffin et al., 2016; Trevisan 2016). As such, this finding may result from the use of self-report measures, with younger children being more likely to overestimate their competence.

Little work has compared parent and self-report measures of emotional self-awareness. In Griffin and colleagues’ (2016) study, neurotypical children had higher self-reported alexithymia compared to parent-reported alexithymia. By comparison, parents of autistic children made similar alexithymia ratings compared to their children. This suggests that parents of typical children may overestimate their child’s abilities, whereas autistic children and their parents tend to make similar estimates. If this is the case, the area of inconsistency lies in neither the autistic child nor their parent, but in lack of agreement between neurotypical children and their parents. This further demonstrates the need for research utilising both parent- and self-report measures.

The lack of behavioural tools to assess emotional self-awareness make it difficult to make sense of these findings. Without an objective point of comparison, it is difficult to assess whether children overestimate their abilities, or whether parents under-estimate them. Parents in these studies will have full knowledge of their child’s diagnostic status and the associated stereotypes may influence parent-report outcomes. Furthermore, autistic children may be less likely to communicate their own emotional states to peers and adults, due to either communication difficulties or anxiety, leading to the misconception they lack self-awareness of them. A key implication of our review is that future research should attempt to implement objective measures alongside other-report and self-report measures. This will allow researchers to examine whether emotional self-awareness difficulties in autism result from report-bias, as well as when difficulties emerge during development.

A further limitation of our review is the relatively limited age range of the data set. Very few studies have examined autism beyond young adulthood, making it difficult to assess how this developmental trajectory may change in later life. Previous research has found that emotional self-awareness becomes worse with increasing age in the general population (Mattila et al., 2006) – such an effect may be particularly negative in autism.

Finally, there was a limited number of studies examining emotional self-awareness in pre-adolescent children. This likely links to the reliance on self-report measures in the wider literature, as such methods are often not suitable for very young children, particularly in autistic groups, where verbal and cognitive delays are common. As a result, our current findings are based on a relatively small pool of studies for children of age 12 years and below. Future research may benefit from finding more ways to examine this key psychological construct in young samples.

We found that while emotional self-awareness was diminished in autism across different measurement tools, these difficulties do not seem to be present in early childhood. Instead, emotional self-awareness difficulties emerge during adolescence and worsen with age. However, our findings are limited by the reliance on self-report measures in the literature. Future research would benefit from including observer-report and behavioural methods of measuring emotional self-awareness, to account for self-bias. Finally, our review suggests that adolescence may be a critical period for emotional self-awareness development in autism. Targeting emotional self-awareness in autistic children may protect against the increasing emotional and social challenges of adolescence.

## Supplemental Material

Supplementary_Information_A – Supplemental material for Emotional self-awareness in autism: A meta-analysis of group differences and developmental effects Click here for additional data file. Supplemental material, Supplementary_Information_A for Emotional self-awareness in autism: A meta-analysis of group differences and developmental effects by Charlotte F Huggins, Gemma Donnan, Isobel M Cameron and Justin HG Williams in Autism

Supplementary_Information_B – Supplemental material for Emotional self-awareness in autism: A meta-analysis of group differences and developmental effects Click here for additional data file. Supplemental material, Supplementary_Information_B for Emotional self-awareness in autism: A meta-analysis of group differences and developmental effects by Charlotte F Huggins, Gemma Donnan, Isobel M Cameron and Justin HG Williams in Autism
