# Supplementary material for: Emotional self-awareness in autism: A meta-analysis of group differences and developmental effects
Source: Autism. 2020 Nov 5;25(2):307–21. doi: 10.1177/1362361320964306 (PMC7874376; doi:10.1177/1362361320964306)
Supplement: Supplementary_Information_A – Supplemental material for Emotional self-awareness in autism: A meta-analysis of group differences and developmental effects [file Supplementary_Information_A.docx]

**Supplementary Information A**

## Risk of Bias

### Quality Assessment

Out of 47 studies, 41 controlled for participant age, 41 for participant gender, and 26 for IQ, level of education or language ability. All but 3 studies indicated that clinical participants had autism diagnoses from independent clinicians. Of these, 8 studies used both ADI and ADOS to verify diagnosis, 10 used only the ADOS, 4 used only the ADI, and the remaining 22 did not use ADI or ADOS.

Few studies controlled for comorbid mental health issues. Only 12 of the total 47 records controlled for the impact of mental health issues on emotional self-awareness, such as through including symptoms as a covariate or excluding participants with any comorbid mental health conditions. 8 studies examined the relationship between autism symptom severity and emotional self-awareness.

Comparing recruitment methods, 12 studies showed completely comparable recruitment methods for autistic and non-autistic participants. 11 studies showed some overlap between recruitment methods for the different participant samples. In the remaining 24 studies, participants with and without autism were recruited from completely different sources. In the 35 studies with different or partially overlapping recruitment methods, 19 (54%) recruited participants with autism from clinical settings, such as clinics and special education centres. In 9 studies (25%) recruitment routes were unclear.

### Publication Bias

A funnel plot of the standard mean difference between groups across all studies was created, see figure A. Six studies were excluded from this funnel plot (Boily, Kingston, & Montgomary, 2017; Duijkers et al., 2014; Karbasdehi et al., 2018; Lai et al., 2013; Rieffe et al., 2007) as average total emotional self-awareness scores were not available. All data was coded so that higher scores represented lower emotional self-awareness.

Funnel plots were asymmetrical, suggesting a possibility of publication bias, with findings that show less difference between groups being less likely to reach publication.

**Overall Risk of Bias**

Overall, quality of the research included in the current analysis in relation to our research question was fair. Consistent issues included the lack of mental health outcomes as a covariate, although as the majority of studies were not explicitly addressing differences in granularity as a research question, this is to be expected. There was some suggestion of publication bias. However, as emotional self-awareness was often not the main outcome of interest in the studies, and thus unlikely to influenced publisher’s decisions, this bias is unlikely to be particularly strong. We judged that the current body of research was of good enough quality and presented little enough risk of bias to make meaningful comparisons in the current review


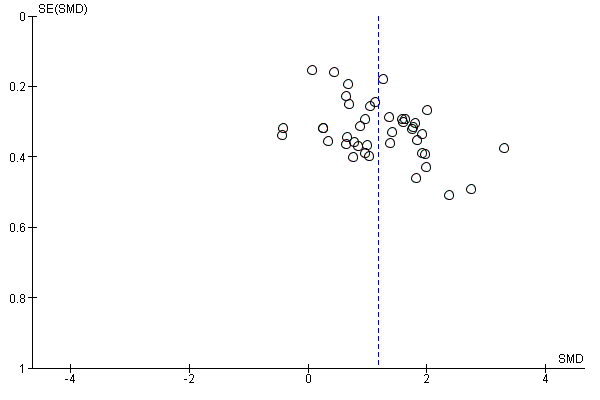


**Figure A.** Funnel plot of all included studies.
