# Supplementary material for: Emotional self-awareness in autism: A meta-analysis of group differences and developmental effects
Source: Autism. 2020 Nov 5;25(2):307–21. doi: 10.1177/1362361320964306 (PMC7874376; doi:10.1177/1362361320964306)
Supplement: Supplementary_Information_B – Supplemental material for Emotional self-awareness in autism: A meta-analysis of group differences and developmental effects [file Supplementary_Information_B.docx]

# Supplementary Information B

| **Table 1**  Summary table of all studies included in dataset. | | | | | | | | | | | | |
| --- | --- | --- | --- | --- | --- | --- | --- | --- | --- | --- | --- | --- |
| **#** | **Authors** | **Year** | **Participants [ASD/NT]** | **No. of Females [ASD/NT]** | **Age Group** | **Term** | **Measure Type** | **Measure** | **Granularity lower in autism?** | **P-Value** | **Effect size** | **Quality [Max: 19]** |
| 1 | Murray et al. | 2017 | 20/20 | 0/1 | Adults | Alexithymia | Self-report | TAS-20 | **Yes** | p < .001 | d = 1.41 | 8 |
| 2 | Milosavljevic et al. | 2016 | 56/32 | 2/0 | Adolescents | Alexithymia | Self-report | TAS-20 | **Yes** | P < .01 | NR | 15 |
| 3 | Griffin, Lombardo & Auyeung | 2016 | 25/32 | 2/17 | Children | Alexithymia | Self-report; Parent-Report | CAQ-SR; CAM | **Yes** | p = .009 p < .0001 | d = .94 d = 1.74 | 12 |
| 4 | Trevisan, Bowering, & Birmingham | 2016 | 17/17 | 4/4 | Children | Alexithymia | Parent-report | CAM | **Yes** | p = .006 | d = 1.02 | 10 |
| 5 | Tani et al | 2004 | 20/10 | 6/3 | Adults | Alexithymia | Self-report | TAS-20 | **Yes** | p < .001 | NR | 8 |
| 6 | Rieffe et al. | 2007 | 22/22 | 2/2 | Children | Awareness of multiple emotions | Behavioural task | Multiple Emotions Task | **Yes** | p = .037 | NR | 5 |
| 7 | Hill, Berthoz, & Frith * | 2004 | 27/74 | 12/46 | Adults | Emotional processing and reflection | Self-report | TAS-20 | **Yes** | p < .001 | NR | 7 |
| 8 | Brady et al | 2014 | 34/34 | 8/8 | Adults | Intra-personal emotional intelligence | Self-report | Bar-On EQ-i | **Yes** | p < .01 | d = .71 | 7 |
| 9 | Patil et al. | 2016 | 17/17 | 6/4 | Adults | Alexithymia | Self-report | TAS-26 | **Yes** | p < .001 | NR | 12 |
| 10 | Roberts-Collins et al. | 2017 | 56/56 | 19/19 | Adolescents | Emotional awareness | Self-report | EAQ | **Yes** | p = .001 | NR | 5 |
| 11 | Erbas et al. | 2013 | 18/18 | 6/6 | Adolescents | Emotion differentiation | Behavioural task | Photo Emotion Differentiation Task; Emotion Sorting Task | *Mixed* | p = .04 p = .06 | NR | 7 |
| 12 | Ketelaars et al. | 2016 | 31/28 | 31/28 | Adults | Alexithymia | Self-report | BVAQ | **Yes** | p < .001 | d = 1.63 | 7 |
| 13 | Rieffe et al. | 2011 | 66/118 | 8/14 | Children | Emotional awareness | Self-report | EAQ | NR | NR | NR | 9 |
| 14 | Samson, Huber & Gross | 2012 | 27/27 | 16/16 | Adults | Emotion labelling | Self-report | TAS-20 | **Yes** | p < .001 | ᶯ = .66 | 10 |
| 15 | Hagenmuller et al | 2014 | 29/28 | 11/11 | Adults | Alexithymia | Self-report | TAS-20 | **Yes** | p < .001 | d = 1.68 | 3 |
| 16 | Schneider et al. | 2013 | 28/28 | 13/13 | Adults | Alexithymia | Self-report | TAS-20 | **Yes** | p < .001 | NR | 10 |
| 17 | Costa, Steffgen, & Samson | 2017 | 37/41 | 5/9 | Children | Alexithymia | Parent-report | Alexithymia Questionnaire for Children | **Yes** | p < .001 | ᶯ = .34 | 5 |
| 18 | Berthoz et al. ** | 2013 | 38/134 | 14/81 | Adults | Alexithymia | Self-report | TAS-20; BVAQ | **Yes** | P < .001 | ᶯ = .30 | 10 |
| 19 | Courty et al. | 2013 | 15/15 | 2/2 | Adults | Alexithymia | Self-report | BVAQ | **Yes** | p = .014 | NR | 9 |
| 20 | Silani et al. | 2008 | 15/15 | 2/2 | Adults | Emotional awareness | Self-report | TAS-20; BVAQ | *Mixed* | p < .01 p > .05 |  | 6 |
| 21 | Heaton et al. | 2012 | 20/20 | 5/5 | Adults | Alexithymia | Self-report | TAS-20 | NR | NR | NR | 8 |
| 22 | Maisel et al. | 2016 | 76/75 | 17/17 | Adults | Alexithymia | Self-report | TAS-20 | *Subscales addressed only* | DIF < .001 DDF < .001 | NR | 13 |
| 23 | Brezis et al | 2017 | 34/35 | 3/7 | Adults | Alexithymia | Self-report | TAS-20 | **Yes** | p < .001 | NR | 7 |
| 24 | McCrimmon, Matchullis, & Altomare | 2016 | 20/20 | 1/1 | Children | Intra-personal emotional intelligence | Self-report | Bar-On EQ-i | No | p = .178 | NR | 7 |
| 25 | Lombardo et al. | 2007 | 30/30 | 7/7 | Adults | Self-awareness | Self-report | TAS-20 | **Yes** | p < .001 | d = 1.40 | 9 |
| 26 | Dijkhuis et al. *** | 2017 | 75/38 | 8/5 | Adults | Emotional processing | Self-report | BVAQ | **Yes** | p < .05 | NR | 10 |
| 27 | Duijkers et al. | 2014 | 51/53 | 5/5 | Adults | Social cognition | Self-report | BVAQ; Bar-On EQ-i | **Yes** | p < .001 | NR | 4 |
| 28 | Allen, Davis, & Hill † | 2013 | 23/24 | 5/6 | Adults | Alexithymia | Self-report | BVAQ; TAS-20 | NR | NR | NR | 6 |
| 29 | McCrimmon, Climie, & Huynh | 2017 | 18/18 | 0/0 | Children | Alexithymia | Self-report | Bar-On EQ-i | No | p > .05 | NR | 8 |
| 30 | Kopec, Hillier, & Frye | 2014 | 24/43 | 2/7 | Adults | Alexithymia | Self-report | TAS-20 | NR | NR | NR | 5 |
| 31 | Berthoz & Hill | 2005 | 19/29 | 6/18 | Adults | Alexithymia | Self-report | TAS-20; BVAQ | *Mixed* | p < .01 p = .17 | NR | 7 |
| 32 | Boily, Kingston, & Montgomery | 2017 | 25/25 | 7/5 | Adolescents | Intra-personal emotional intelligence | Self-report | Bar-On EQ-i | No | p = .225 | NR | 5 |
| 33 | Thaler et al. | 2018 | 16/16 | 0/0 | Adults | Alexithymia | Self-report | TAS-20 | No | p = .33 | NR | 6 |
| 34 | Arellano et al | 2017 | 14/21 | 0/0 | Adolescents | Alexithymia | Self-report | TAS-26; BVAQ | *Mixed* | p = .030; p = .380 | g = .95; g = .32 | 2 |
| 35 | Bernhardt et al. | 2014 | 16/16 | 4/7 | Adults | Alexithymia | Self-report | TAS-20 | **Yes** | p = .07 | d = .65 | 8 |
| 36 | Brewer et al. | 2016 | 14/13 | 1/0 | Adults | Alexithymia | Self-report | TAS-20 | NR | NR | NR | 9 |
| 37 | Brewer et al. | 2017 | 19/27 | 3/8 | Adults | Alexithymia | Self-report | TAS-20 | **Yes** | P = .005 | NR | 8 |
| 38 | Fan et al. | 2014 | 34/35 | 0/0 | Adults | Alexithymia | Self-report | TAS-20 | **Yes** | P < .001 | NR | 8 |
| 39 | Gu et al. | 2016 | 17/17 | 0/0 | Adults | Alexithymia | Self-report | TAS-20 | **Yes** | P < .001 | NR | 9 |
| 40 | Katsyri et al. | 2008 | 20/20 | 7/7 | Adults | Alexithymia | Self-report | TAS-20 | **Yes** | P < .001 | NR | 8 |
| 41 | Krach et al | 2015 | 16/16 | 0/0 | Adults | Alexithymia | Self-report | TAS-20 | No | P = .059 | NR | 9 |
| 42 | Lai et al | 2013 | 60/60 | 30/30 | Adults | Self-awareness of own emotions | Self-report | TAS-20 | **Yes** | P < .001 | NR | 12 |
| 43 | Lombardo et al | 2010 | 29/33 | 0/0 | Adults | Alexithymia | Self-report | TAS-20 | **Yes** | P < .001 | NR | 8 |
| 44 | Minio-Paluello et al. | 2009 | 14/14 | 0/0 | Adults | Alexithymia | Self-report | TAS-20 | **Yes** | p < .001 | NR | 8 |
| 45 | Karbadeshi, Abolghasemi & Karbasdehi | 2018 | 60/60 | 0/0 | Adolescents | Alexithymia | Self-report | TAS-20 | *Subscales addressed only* | DIF,  p = .039  DDF, p = .003  EOT,  p = .001 | DIF  η = .072  DDF  η = .142 EOT  η = .487 | 3 |
| 46 | Wieckowski & White | 2017 | 20/20 | 6/11 | Adolescents | Alexithymia | Self-Report | CAQ-SR | NR | NR | NR | 8 |
| 47 | Mul et al. | 2018 | 26/26 | 7/7 | Adults | Alexithymia | Self-report | TAS-20 | **Yes** | P = .001 | d = .97 | 7 |
| ***Note:*** ASD: Autism Spectrum Disorder. NT: Neurotypical (non-autistic). TAS-20: 20-item Toronto Alexithymia Scale. CAQ-SR: Children’s Alexithymia Questionnaire Self-Report. CAM: Children’s Alexithymia Measure. Bar-On EQ-i: Bar-On Emotional Quotient Inventory. TAS-26: 26-item Toronto Alexithymia Scale. BVAQ: Bermond-Vorst Alexithymia Questionnaire. EAQ: Emotional Awareness Questionnaire. DIF: Difficulty Identifying Feelings TAS-20 subscale. DDF: Difficulty Describing Feelings TAS-20 subscale. EOT: Externally-Oriented Thinking TAS-20 subscale. NR: “Not Reported”  † This paper did not report the use of the TAS-20 in publication, but did collect unpublished data. This data was shared with CH.  * Full control sample 74 included relatives of autistic adults. The 35 typical adult controls with no autistic relatives used for analysis.  ** Full control sample 136 included parents of autistic children. 47 typical adult controls with no autistic relatives used for analysis.  *** Means and standard deviations received directly from authors, number of participants differed from that reported in paper. Data from authors used in analysis. | | | | | | | | | | | | |

| **Table 2**  Measurement tools frequency and summaries, first published in *Huggins, Donnan, Williams & Cameron (in press)* | | | | |
| --- | --- | --- | --- | --- |
| Measurement Tool | Type | Frequency | Target Population | Description |
| Toronto Alexithymia Scale (20 item)  [TAS-20] | Self-report | 29 † | Adults | 20-item self-report composed of three factors: difficulty identifying feelings (DIF), difficulty describing feelings (DDF) and externally oriented thinking (EOT). Intended to assess alexithymia, including tendency to focus on external over internal stimuli. Responses on 5-point scale, summed to calculate total. Some items reverse-scored.  Good overall internal consistency and high test-rest reliability (Bagby, Parker, & Taylor, 1994), but EOT tends to have unsatisfactory internal consistency (Kooiman, Spinhoven, & Trijsburg, 2002)  Scores range from 20 to 100.  Higher scores represent lower emotional self-awareness. |
| Bermond-Vorst Alexithymia Questionnaire  [BVAQ] | Self-report | 7 | Adults | Self-report questionnaire with a five-factor structure, organised into two components: cognitive and affective alexithymia (Vorst & Bermond, 2001). Responses on 5-point scale, summed to calculate total. Half of the items reverse-scored.  Cognitive component composed of ‘Identifying emotions’, ‘Analysing emotions’ and ‘Verbalizing emotions’ subscales. Affective component composed of ‘Emotionalizing’ and ‘Fantasising’ subscales.  Three versions of the BVAQ in use: 40-item BVAQ-AB, 20-item BVAQ-A, and 20-item BVAQ-B. BVAQ-A and BVAQ-B “parallel” forms, measuring same factors with same number of items.  BVAQ-AB scores range from 40 to 200. BVAQ-A and BVAQ-B scores range from 20 to 100.  Higher scores represent lower emotional self-awareness. |
| Bar-On Emotional Quotient Inventory Intrapersonal Subscale  [Bar-On EQ-i] | Self-report | 5 | Any | Bar-On EQ-I interpersonal scale is subscale of the Bar-On EQ-I, self-report measure of emotional intelligence.  Intrapersonal subscale assesses ability to be aware of, express and assess own emotions, as well as self-efficacy and overall positive affect. Intrapersonal subscale scores significantly correlate with TAS-20 scores (Dawda & Hart, 2000)  Four versions of the EQ-I exist: the 133 item adult form, the 51 item adult form, the 60 item short youth form, and the 30 item short adult form.  Higher scores represent higher emotional self-awareness. |
| Toronto Alexithymia Scale (26 item)  [TAS-26] | Self-report | 2 | Adults | A 26-item, four-factor version of the TAS-20, including the DIF, DDF, and EOT subscales alongside the subscale ‘Reduced daydreaming’ (RD). Largely out of use in English speaking world, but still used in some countries where the TAS-20 has not been well-validated.  Internal consistency tends to be good, aside from in the EOT subscale (Kauhanen, Julkunen, & Salonen, 1992; Lovko, Gelo, & Karlović , 2015)  Higher scores represent lower emotional self-awareness. |
| Emotional Awareness Questionnaire – Differentiation Subscale  [EAQ] | Self-report | 2 | Adults | 7-item subscale of the six-factor, 30-item EAQ. Responses on three point scale, averaged to calculate total.  EAQ-diff shows moderate internal reliability in both child (a = .67) and adolescent (a = .74) populations (Rieffe et al., 2008).  Higher scores represent higher emotional self-awareness. |
| Children’s Alexithymia Measure  [CAM] | Other-report | 2 | Children | 14-item parent-report questionnaire, intended for use in children age 5 to 17. 4-point scale, summed to calculate total. Some items reverse-scored.  High internal reliability (a = .92) and good concurrent validity (r = .73) with the Alexithymia Scale for Children (Way et al., 2010).  Higher scores represent lower emotional self-awareness. |
| Children’s Alexithymia Questionnaire  [CAQ] | Self-report | 2 | Children | 20-item self-report measure based on the TAS-20, with identical 3-factor structure. Responses on 3-point scale, summed to calculate total. Some items reverse-scored.  Internal reliability high for DIF (a = .73) and DDF (a = .75) subscales, but poor for EOT (a = .29) subscales (Rieffe, Oosterveld, & Terwogt, 2006).  Higher scores represent lower emotional self-awareness. |
| Multiple Emotions Task  [MET] | Behavioural task | 1 | Children | Children present with four stories of emotional events, and asked to imagine the story happened to them. Two stories designed to evoke positive and negative emotion, two designed to evoke different negative emotions.  Children indicate how they would feel in each instance by pointing to four facial expressions (sad, angry, happy, scared), and rating intensity of hypothetical feeling on five-point scale.  Number of emotional perspectives identified taken to reflect greater awareness of multiple emotions.  High scores represent higher emotional self-awareness. |
| Photo Emotion Differentiation Task  [PED-task] | Behavioural task | 1 | Any | Participants view 20 negative emotional images, selected to match 20 discrete negative emotions. Participants how intensely they experienced 20 emotions in response to each image.  Intra-class correlation coefficients of consistency calculated between emotion ratings. Higher ICCs reflect less differentiation between emotion terms.  ICC scores significantly negatively correlated with DIF subscale of TAS-20, r = .32, p < .05, although no other correlation between PED-task and TAS-20 scores found (Erbas et al., 2014). Showed no significant correlations with the ES-task (Erbas et al., 2013).  High scores represent lower emotional self-awareness. |
| Emotion Sorting Task  [ES-task] | Behavioural task | 1 | Any | 20 negative emotion terms printed on cards, and participants asked to sort them into groups based on how similar they perceived the emotions to be. More groups indicated greater ability to differentiate between emotions.  Showed no significant correlations with the PED-task (Erbas et al., 2013)  High scores represent higher emotional self-awareness. |
| Alexithymia Questionnaire for Children  [AQC] | Other report | 1 | Children | 20-item parent report measure, based upon the CAQ, for use in children between 5 to 17 years old. Has identical three-factor model to the TAS-20. Responses on three-point scale, summed to produce total scores.  Internal consistency satisfactory in both French (a = .73) and German (a = .86) versions of the questionnaire (Costa, Steffgen, & Samson, 2017).  Higher scores represent lower emotional self-awareness. |
| Note. Some tools appear in the same paper.  † One paper did not report the use of the TAS-20 in publication, but did collect unpublished data. This data was shared by the authors with CH. | | | | |

**Table 2 Citations**

Bagby, R. M., Parker, J. D. A., & Taylor, G. J. (1994) The Twenty-item Toronto Alexithymia Scale – I. Item selection and cross-validation of the factor structure. Journal of Psychosomatic Research, 38(1), 23 – 32. doi: 10.1016/0022-3999(94)90005-1

Costa, A. P., Steffgen, G., & Samson, A. C. (2017) Expressive Incoherence and Alexithymia in Autism Spectrum Disorder. *Journal of Autism and Developmental Disorders, 47,* 1659 – 1672. doi: 10.1007/s10803-017-3073-9

Dawda, D., & Hart, S. D. (2000) Assessing emotional intelligence: reliability and validity of the Bar-On Emotional Quotient Inventory (EQ-i) in university stuednts. *Personality and Individual Differences, 28*(4), 797 – 812. doi: 10.1016/S0191.8869(99)00139-7

Erbas, Y., Ceulemans, E., Boonen, J., Noens, & Kuppens, P. (2013) Emotion differentiation in autism spectrum disorder. *Research in Autism Spectrum Disorders, 7,* 1221 – 1227. doi: 10.1016/j.rasd.2013.07.007

Erbas, Y., Ceulemans, E., Pe, M. L., Koval, P., & Kuppens, P. (2014) Negative emotion differentiation: Its personality and well-being correlates and a comparison of different assessment methods. *Cognition and Emotion, 28*(7), 1196 – 1213. doi: 10.1080/02699931.2013.875890

Kauhanen, J., Julkunen, J., & Salonen, J. T. (1992) Coping with inner feelings and stress: Heavy alcohol use in the context of alexithymia. *Behavioral Medicine, 18*(3), 121 – 126. doi: 10.1080/08964289.1992.9936962

Kooiman, C. G., Spinhoven, P., Trijsburg, R. W. (2002) The assessment of alexithymia: A critical review of the literature and a psychometric study of the Toronto Alexithymia scale-20. *Journal of Psychosomatic Research, 53,* 1083 – 1090. doi: 10.1016/s0022-3999(02)00348-3

Lovko, S. K., Gelo, J., & Karlović, D. (2015) Validation study of the Toronto Alexithymia Scale (TAS-26) in Croatian Population. *Acta Clinica Croatica, 54*(3), 272 – 277.

Rieffe, C., Oosterveld, P., & Terwogt, M. M. (2006) An alexithymia questionnaire for children: Factorial and concurrent validation results. *Personality and Individual Differences, 40,* 123 – 133. doi: 10.1016/j.paid.2005.05.013

Rieffe, C., Oosterveld, P., Miers, A. C., Terwogt, M. M., & Ly, V. (2008) Emotion awareness and internalising symptoms in children and adolescents: The Emotion Awareness Questionnaire revised. *Personality and Individual Differences, 45*(8), 756 – 761. doi: 10.1016/j.paid.2008.08.001

Vorst, H. C. M., & Bermond, B. (2001) Validity and reliability of the Bermond-Vorst Alexithymia Questionnaire. *Personality and Individual Differences, 30,* 413 – 434. doi: 10.1016/S0191-8869(00)00033-7

Way, I. F., Applegate, B., Cai, X., Franck, L. K., Black-Pond, C., Yelsma, P., Roberts, E., Hyer, Y., & Muliett, M. (2010) Children’s Alexithymia Measure (CAM): A New Instrument for Screening Difficulties with Emotional Expression. *Journal of Child & Adolescent Trauma, 3*(4), 303 – 318. doi: 10.1080/19361521.2011.609772

| Quality Assessment | | |
| --- | --- | --- |
| Citation |  | |
| Overall Score | \| 0 \| 1 \| 2 \| 3 \| 4 \| 5 \| 6 \| 7 \| 8 \| 9 \| 10 \| 11 \| 12 \| 13 \| 14 \| 15 \| 16 \| 17 \| 18 \| 19 \| \| --- \| --- \| --- \| --- \| --- \| --- \| --- \| --- \| --- \| --- \| --- \| --- \| --- \| --- \| --- \| --- \| --- \| --- \| --- \| --- \| \|  \|  \|  \|  \|  \|  \|  \|  \|  \|  \|  \|  \|  \|  \|  \|  \|  \|  \|  \|  \| \| Poor \| \| \|  \|  \|  \|  \|  \| Good \| \| \| \|  \|  \|  \|  \|  \| Excellent \| \| \| | |
| Recruitment | | |
| ASD Recruitment Method | | General population  Clinical population |
| *Details* | |  |
| TD Recruitment Method | | General population  Clinical population |
| *Details* | |  |
| Recruitment Comparability | | No comparability  Some overlap  Completely comparable |
|  | | *+ 0 +1 +2* |
| Sample Bias | | |
| Sample matched on | | Age  IQ  Gender  *+ 1 +1 +1* |
| ASD Diagnosis | | |
| Diagnosis method | | Independent clinician  Self-diagnosis  Experimenters  Unclear |
|  | | *+ 2 +0 +0 +0* |
| Severity assessment | | ADOS  ADI  Other  *+ 1 +1 +1 (or appropriate)* |
| Severity compared to granularity | | Yes  No  Unclear |
|  | | *+ 1 +0 +0* |
| *Details* | |  |
| Confounding Factors | | |
| Measured | | Depression  Anxiety  Any other distress  Autistic traits  *+ 1 +1 +1 +1* |
| Controlled for | | Depression  Anxiety  Any other distress  Autistic traits |
|  | | *+ 1 +1 +1 +1* |
| *Details* | |  |
| Other Factors | | |
| *Details* | |  |
| **Figure 1:** Quality assessment form used in data extraction. | | |
